# Role of Short-Chain Fatty Acids Produced by Gut Microbiota in Innate Lung Immunity and Pathogenesis of the Heterogeneous Course of Chronic Obstructive Pulmonary Disease

**DOI:** 10.3390/ijms23094768

**Published:** 2022-04-26

**Authors:** Stanislav Kotlyarov

**Affiliations:** Department of Nursing, Ryazan State Medical University, 390026 Ryazan, Russia; SKMR1@yandex.ru

**Keywords:** COPD, innate immune system, gut microbiota, short-chain fatty acids, nutritional support, phenotype

## Abstract

Chronic obstructive pulmonary disease (COPD) is a widespread socially significant disease. The development of COPD involves the innate immune system. Interestingly, the regulation of the innate lung immune system is related to the gut microbiota. This connection is due to the production by gut microorganisms of short-chain fatty acids (SCFAs) such as acetate, propionate, and butyrate. Nutritional disturbances and changes in the structure of the intestinal microbiota lead to a decrease in SCFAs production and their effect on pulmonary immunity. The presence of a metabolic and immune axis linking the lungs and gut plays an important role in the pathogenesis of COPD. In addition, the nature of nutrition and SCFAs may participate in the development of the clinically heterogeneous course of COPD.

## 1. Introduction

Chronic obstructive pulmonary disease (COPD) is one of the most common respiratory diseases of social significance [1]. It is an important cause of health care seeking, hospitalizations, and disability [2,3]. Epidemiological studies show unfavorable trends in the prevalence of COPD in many countries [4,5]. The urgency of the problem is also increased by the fact that COPD is among the leading causes of death in the world. Moreover, treatment and rehabilitation of patients remain an unsolved problem in many respects, which requires new more detailed data on pathophysiological mechanisms of disease development and on those parts of pathogenesis, which can be effectively influenced for therapeutic purposes.

The development and progression of the disease is associated with exposure to tobacco smoke components and exogenous aeropollutants [6,7,8]. Inflammation in the bronchial tree is known to underlie the pathogenesis of COPD and is characterised by the involvement of many cells, including macrophages and neutrophils [9]. Prolonged persistent inflammation is associated with impaired immune mechanisms that provide a balance between maintaining and resolving inflammation [10].

It is known that COPD is a disease characterized by both pulmonary and extrapulmonary clinical heterogeneity [11,12]. Moreover, some features of the clinical picture and phenotypes of COPD are related to nutrition. Body mass index (BMI) is considered as one of the important markers in assessing the prognosis of COPD [13]. Lower body weight is associated with a greater risk of adverse outcome, whereas excess body weight has an even better prognosis [14]. This phenomenon has been called the “obesity paradox” [15,16,17]. The course of COPD, in which higher energy requirements lead to loss of fat and muscle tissue, is thought to be prognostically unfavorable [18,19]. The increased energy requirement may be related both to respiration and to the maintenance of inflammation in the bronchi. Dietary modification is considered an important tool that can improve the clinical course of COPD [20]. It is suggested that the nature of nutrition may be related to the pathophysiological mechanisms of COPD not only through the influence on energy and metabolic processes, but also through the regulation of some immune mechanisms [21].

A growing body of evidence supports a metabolic and immune axis linking the gut and lungs [22,23]. These links are bidirectional, and the gut microbiome is one of the central links in this interaction. The gut is the site of localization of much of the commensal bacterial mass [24,25]. This microbiome is metabolically active, participating in the production of many substances, such as, short-chain fatty acids (SCFAs) [26]. Accumulating evidence suggests that SCFAs can be considered as a leading link in the metabolic and immune axis between the gut and lungs. Indeed, SCFAs exhibit a variety of functions in immune defence, making them a potentially important target for clinical and experimental research.

Thus, the aim of this review is to discuss the putative role of SCFAs produced by intestinal microbiota in innate lung immune defence and the pathogenesis of the heterogeneous course of COPD.

## 2. Short-Chain Fatty Acids

### 2.1. Production of SCFAs

SCFAs are straight- or branched-chain fatty acids with less than six carbon atoms. The most common SCFAs are acetate, propionate, and butyrate, which are produced by the intestinal microbiota through anaerobic fermentation of non-digestible polysaccharides. The rate of production, amount, and ratio of SCFAs depend on several factors, such as the species structure and quantitative composition of the microbiota in the colon, the nature of the substrate, and the time of its passage through the gut [27].

Substrates for SCFA formation are dietary fibers, including resistant starch, cellulose, and pectin [28]. Dietary fibers that are not enzymatically processed by humans become a substrate for the microbiota of the large intestine. The colon microbiota hydrolyze undigested carbohydrates into oligosaccharides and then into monosaccharides, followed by fermentation through glycolysis and the pentose phosphate pathway [29].

**Figure 1:**
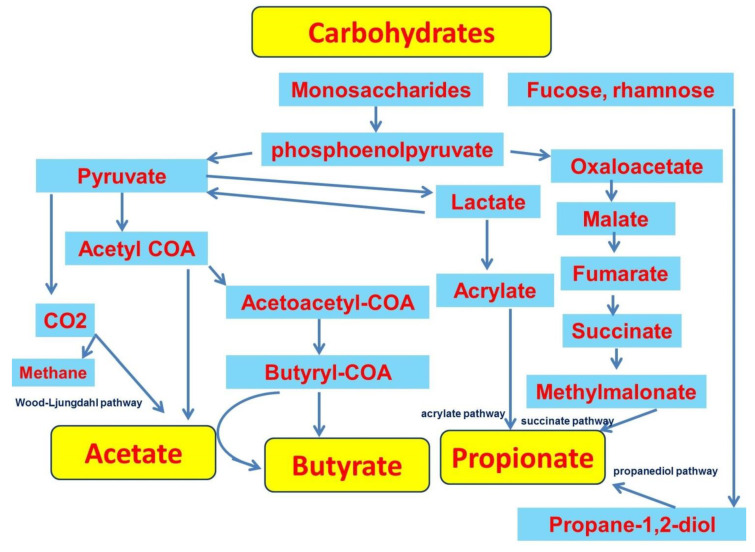
Short-chain fatty acids formation pathways.

Acetate is the major SCFA in the colon, which is due to the widespread occurrence of acetate production pathways among bacteria [30]. Acetate is formed from pyruvate via acetyl-CoA or via the Wood-Ljungdahl pathway [29]. Butyrate is produced by condensation of two acetyl-CoA molecules to form acetoacetyl-CoA followed by reduction to butyryl-CoA [31]. In this process, butyrate production is mediated by butyrate kinase. The other pathway of butyrate formation, which uses acetate of exogenous origin, involves butyryl-CoA:acetate CoA-transferase [29,32]. This variant involves coordination between acetate-producing and butyrate-producing bacteria [33]. Propionate production can occur via the succinate pathway or the acrylate pathway, in which lactate is reduced to propionate, and via the propanediol pathway from deoxy sugars such as fucose and rhamnose (Figure 1) [29,31].

In addition to carbohydrates, SCFA formation in the gut can also occur through amino acid metabolism [31]. Protein fermentation is associated with the formation of branched-chain SCFAs. For example, isobutyrate, 2-methylbutyrate, and isovalerate can be formed from branched-chain amino acids such as valine, isoleucine, and leucine [34]. In addition to fatty acid formation, amino acid fermentation produces potentially harmful metabolites such as phenolic and indole compounds, amines, and ammonia [31,34,35].

### 2.2. SCFAs Transport

SCFAs are found in the largest concentrations in the large intestine, ranging from about 70 to 130 mmol/kg [36,37]. Acetate, propionate, and butyrate are found in the colon in a molar ratio of approximately 57:22:21 [29,37]. SCFAs enter the systemic bloodstream via passive diffusion or involving specific transporters, such as the monocarboxylate transporter 1 (MCT1) and sodium-linked monocarboxylate transporter 1 (SMCT1) [28]. SCFAs are transported through the colonocytes, where most of the butyrate produced is used as the main energy source [38]. Colonocytes can obtain up to 60–70% of their energy from SCFA by β-oxidation and metabolism in the tricarboxylic acid cycle (TCA cycle) [28,29,39,40]. The remaining SCFA fractions enter the portal bloodstream through the basolateral membrane. In the portal vein, the ratio of acetate, propionate, and butyrate is approximately 69:23:8 [29]. In turn, propionate, passing through the portal vein, is metabolized in the liver, where it can be used for gluconeogenesis [41,42]. Because of these processes, acetate is the most common SCFA, as most of it enters the systemic bloodstream. As a result, plasma concentrations of acetate, propionate, and butyrate are approximately 25–250 μmol/l, 1.4–13.4 μmol/l, and 0.5–14.2 μmol/l, respectively [37,43]. The higher plasma levels of acetate are also related to the fact that acetate may not only be of intestinal origin. It can be produced by fatty acid oxidation and amino acid metabolism, by ketogenesis in hepatocytes, and by ethanol oxidation by microsomal cytochrome P450 enzymes [43,44,45,46].

### 2.3. SCFAs Signal Transduction

SCFAs are thought to exert their action by interacting with the G-protein-coupled receptors GPR43 and GPR41, also known as free fatty acid receptor (FFA)2 and FFA3, respectively [47,48,49,50]. SCFAs differ in their selectivity in activating FFA2 and FFA3 receptors. It has been shown that FFA2 is activated to a greater extent by fatty acids with a shorter chain, whereas FFA3 is characterized by an inverse relationship [51]. In particular, this dependence on the number of carbon atoms for FFA2 can be represented as C2 = C3 > C4 > C5 = C1, and for FFA3 as follows: C3 = C4 = C5 > C2 = C1 [52]. GPR43 is expressed in immune cells, including neutrophils, monocytes, and lymphocytes [47,48,49,53].

Other receptors for SCFAs are GPR109a, which is also known as HCA2 and olfactory receptor 78 (Olfr78) [54,55,56]. The GPR109A receptor is expressed predominantly on adipocytes as well as in immune cells such as neutrophils and macrophages [57,58]. Butyrate slightly activates GPR109A, whereas propionate and acetate do not activate the receptor [59].

In addition to binding to the described receptors, SCFAs exert their action through the inhibition of histone deacetylase (HDAC). Histone acetylation is an important mechanism for controlling gene transcription. During this process, acetyl groups are added to histone tails by histone acetyltransferases (HATs) and removed by (HDACs). HDACs are a class of enzymes that inhibit transcription through the removal of acetyl groups from chromatin [47,60]. Butyrate, being the strongest HDAC inhibitor, alters the expression of many genes with different functions [61,62]. Through HDAC inhibition, SCFAs are involved in the regulation of many cellular functions, including migration and survival [47,63,64,65,66].

### 2.4. Participation of SCFAs in the Regulation of Metabolic and Immune processes

**Figure 2:**
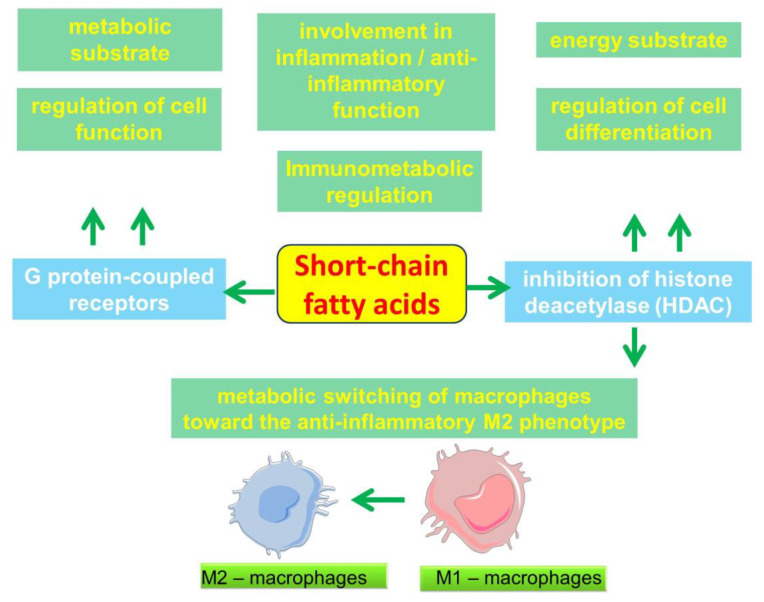
Scheme of the involvement of short-chain fatty acids in cell function and inflammation.

A growing body of evidence strengthens the understanding of the importance of SCFAs in the regulation of some metabolic and immune processes (Figure 2). Through HDAC3 inhibition, butyrate can induce a metabolic switch of macrophages toward an anti-inflammatory M2 phenotype [67,68]. These metabolic and immunological changes in macrophages are in many ways opposite to the well-known proinflammatory M1 activation of macrophages induced by lipopolysaccharide (LPS) stimulation [69]. In the M1 phenotype, macrophages are known to rely primarily on glycolysis as an energy source, which has similarities to the Warburg effect that is characteristic of tumor cells [70]. Although glycolysis is a less efficient energy source, its volume can be rapidly increased, which can rapidly provide energy for the cell. Macrophages differentiated in the presence of butyrate show decreased glycolysis, increased adenosine monophosphate (AMP), increased AMP kinase (AMPK) phosphorylation, and inhibition of the mammalian target of rapamycin (mTOR) [43]. mTOR is a known positive regulator of several enzymes involved in glycolysis, including hexokinase II, glyceraldehyde 3-phosphate dehydrogenase and lactate dehydrogenase-B [71,72]. Inhibition of mTOR may explain the decrease in glycolysis in macrophages that were differentiated in the presence of butyrate [73]. In addition, butyrate-treated macrophages showed an increase in ribulose 5-phosphate, which against the background of decreased intracellular glucose levels indicates an increased flux into the pentose-phosphate pathway, which may contribute to NADPH formation [73]. This effect on cellular metabolism is of particular interest. Thus, by altering metabolic programming in alveolar macrophages, SCFAs may be involved in the regulation of lung immune tone [74].

In addition, by influencing metabolic pathways such as glycolysis enhancement and OXPHOS activity, SCFAs can participate in CD8+ T-cell activation [75]. By influencing cellular metabolism, butyrate has also been shown to promote memory potential in activated CD8+ T cells [76]. These data indicate the involvement of SCFAs in the regulation of distinct parts of both innate and adaptive immunity.

Interestingly, butyrate can act differentially in its effect on cellular metabolism. Butyrate can stimulate the proliferation of normal colonocytes while inhibiting the proliferation of cancerous colonocytes, which are characterized by the Warburg effect in their metabolism. This differential effect is known as the “butyrate paradox” [77]. This effect is due to the fact that butyrate can act as an HDAC inhibitor in cancer cells but as a HAT activator in normal cells.

Macrophages differentiated in the presence of butyrate show increased antimicrobial activity. This is due to the inhibition of histone deacetylase 3 (HDAC3), a shift in macrophage metabolism, decreased mTOR kinase activity, increased LC3-associated host defense and antimicrobial peptide production [73]. The anti-inflammatory effects of butyrate have also been linked to inhibition of the NF-kB signaling pathway as well as production by mononuclear cells and neutrophils of anti-inflammatory cytokines such as IL-10 [62,68,78,79]. In addition to neutrophils, butyrate stimulates the production of IL-10 and inhibits the production of IL -12 and interferon-γ (IFN-γ) by dendritic cells [80].

Another HDAC-related effect of butyrate is the inhibition of nitric oxide (NO) production (via inducible nitric oxide synthase (iNOS)) and proinflammatory cytokines (interleukin (IL)-6, IL-12, IL-1β) induced by LPS [68,79,81]. At the same time, low (0.5–2.5 mM) SCFAs concentrations increased and high (25–50 mM) concentrations decreased iNOS expression in the experiment [82]. Propionate and butyrate were also shown to reduce tumor necrosis factor alpha (TNF-α), cytokine-induced neutrophil chemoattractant-2 (CINC-2αβ), and NO production by LPS-stimulated neutrophils [78]. In addition to cytokines, the anti-inflammatory ability of SCFAs may also be related to the regulation of prostaglandin E2 (PGE2) production [83].

SCFAs such as acetate, propionate, and butyrate increased neutrophil migration by increasing L-selectin expression on neutrophils and releasing CINC-2 alphabeta [84]. At the same time, it has been shown that SCFAs can reduce excessive airway infiltration by neutrophils by reducing levels of C-X-C Motif Chemokine Ligand 1 (CXCL1) produced by lung monocytes and macrophages [75].

SCFAs are involved in regulating the differentiation, recruitment, and activation of neutrophils, dendritic cells, macrophages, and monocytes, as well as T cells [43,54]. Butyrate can affect the differentiation of dendritic cells derived from human monocytes by keeping dendritic cells in a stable, immature stage [80]. SCFAs inhibit the maturation of monocytes, macrophages, and dendritic cells by altering their ability to capture antigens and reducing their ability to produce proinflammatory cytokines such as IL-12 and TNF-α [43,54,62].

SCFAs are also involved in the regulation of inflammation in endothelial cells. Butyrate inhibited TNF-α-induced activation of NF-kB and enhanced the expression of peroxisome proliferator-activated receptor alpha (PPARα) in HUVECs [85]. In addition, butyrate reduced TNF-α-induced expression of vascular cell adhesion molecule 1 (VCAM-1) and Inter-Cellular Adhesion Molecule 1 (ICAM-1) mRNA in these cells [85]. In turn, propionate could also reduce cytokine-induced VCAM-1 and ICAM-1 expression by inhibiting NF-kB activation [86]. In another study, butyrate was shown to increase ICAM-1 and E-selectin expression in vascular endothelium, with a time-dependent effect [87]. It should be noted that ICAM-1 may play an important role in the pathogenesis of emphysema [88]. ICAM-1 is involved in the transendothelial migration of neutrophils during inflammation [89,90]. In this case, higher soluble ICAM-1 was associated with a more rapid progression of emphysema [88].

Thus, SCFAs have anti-inflammatory and immunomodulatory effects [91]. Other evidence suggests that SCFAs can have not only anti-inflammatory but also pro-inflammatory effects on lung cells, which depends on the concentration of SCFAs and the type of cells studied [92]. Interestingly, however, elevated concentrations of SCFAs can induce the release of interleukin IL-8, IL-6, and IL-1β either alone or in combination with toll-like receptor ligands TLR2 and TLR7 [93].

Thus, the accumulated data to date allow SCFAs to be considered as either pro- or anti-inflammatory molecules, depending on cell type as well as on conditions [54]. These and other data have strengthened the understanding of the importance of nutrition in the pathogenesis of COPD, which is based on chronic inflammation characterized by an imbalance between pro- and anti-inflammatory mechanisms.

## 3. Relationship between Gut Microbiota, Diet and Short-Chain Fatty Acid Production

The intestinal microbial community is characterized by considerable diversity, which depends on many external and internal factors, including age and dietary habits. The intestinal microbiota is involved in maintaining the tone of the immune system. Studies in mice, show that a lack of microbiota causes developmental defects in many body systems, including the immune system [94].

Bacteroidetes (such as Bacteroides and Prevotella) and Firmicutes (such as Lactobacillus, Enterococcus, Clostridium and Bacillus) are thought to be common bacteria in the adult gut, whereas Actinobacteria (such as Bifidobacterium) and Proteobacteria (such as Escherichia) are lower [95]. Most Westerners are carriers of high levels of Bacteroides, whereas Prevotella is more common in non-Westerners who consume a plant-rich diet [96].

Bacteroidetes and Firmicutes are mainly localized in the proximal colon and are involved in SCFAs production [97,98,99]. And members of the Bacteroidetes type mainly produce acetate and propionate, while the Firmicutes type produces butyrate [29,100,101].

Given the emerging close relationship between the gut microbiome and lung function, there is increasing evidence of possible abnormalities in gut microflora structure in smoking and COPD [102,103,104,105]. Smokers have a higher proportion of Bacteroidetes and Prevotella and a lower proportion of Firmicutes and Proteobacteria in the gut microbiota compared to nonsmokers [106,107]. In another study, it was shown that stopping smoking led to an increase in microbial diversity and a significant change in gut microbial composition. At the phylum level, Firmicutes and Actinobacteria increased and the proportion of Bacteroidetes and Proteobacteria decreased [105].

In turn, chronic exposure to inhaled particulate matter, which is another important risk factor for COPD, causes gut dysbacteriosis and metabolic disorders in an experimental model in rats [108]. Chronic exposure to particulate matter for 24 weeks induced COPD-like pathological changes and lung inflammation in rats, which were accompanied by decreased abundance and diversity of the gut microbiota and decreased levels of SCFAs [108].

The species diversity of gut microflora and the number of Bacteroides and Bifidobacteria decreases in the elderly [109,110]. In addition, patients with chronic diseases have changes in gut microflora with an increase in a number of harmful bacteria [111].

Overweight and obesity were associated with a change in the ratio of individual SCFAs in favor of propionate [112]. This was consistent with a higher proportion of Bacteroidetes in the total gut bacterial structure in overweight and obese individuals than in lean volunteers [112]. At the same time, the total SCFAs concentration in fecal samples was more than 20% higher in obese than in lean volunteers [112]. In contrast, other studies have shown a reduction in the Bacteroidetes community in obese patients [113,114].

The gut microbiota is closely related to dietary habits. A comparative study of dietary patterns in African and European children showed that a diet high in fiber and low in animal protein was associated with a significant enrichment of Actinobacteria and Bacteroidetes and less Firmicutes and Proteobacteria. This corresponded to a greater production of short-chain fatty acids due to bacterial fermentation of plant fibers [115].

It should be noted that there are connections between the dietary precursors of SCFAs as well as the composition of plasma SCFAs [116,117,118]. This may be due to the fact that different compositions of the gut microflora may differentially utilize various sources of fermentable fiber.

A diet rich in protein has a significant effect on SCFAs production. High-protein and moderate-carbohydrate and high-protein and low-carbohydrate diets have been shown to increase the proportion of branched-chain fatty acids and the concentration of phenylacetic acid and N-nitroso compounds after four weeks. The high-protein and low-carbohydrate diet also decreased the proportion of butyrate in short-chain fatty acid concentrations in feces [119]. This seems important given the need for nutritional support in COPD due to the skeletal muscle hypotrophy that accompanies this reduction in physical activity.

A high-fat diet has been shown to reduce the number of Bacteroidetes [120,121,122]. The fecal microbiota of vegans was enriched for Verrucomicrobia phyla, while it was reduced for Proteobacteria phyla and lactic acid bacteria. And the vegan group had lower amounts of branched-chain fatty acids, such as iso-valerate and iso-butyrate, as well as acetate and propionate [97].

In turn, the consumption of vegetables, fruits, whole-grain cereals, and oily fish may help protect against worsening lung function in adults, especially in male smokers and COPD patients [123]. Among men, high fiber intake has been shown to be inversely related to the incidence of COPD in both current and former smokers [124]. In addition, the decrease in COPD incidence among men, both smokers and ex-smokers, was associated with high fruit and vegetable intake [125]. Interestingly, the reduced risk of COPD in women was associated with a prolonged consumption of fruit only, not vegetables [126]. It should be noted that these studies do not take into account the contribution of individual food components and micronutrients in providing these connections.

Thus, the nature of nutrition may influence not only the structure of the gut microbiota but also the production of SCFAs, potentially affecting immune regulation, which is important in COPD. This reinforces the importance of additional studies that could improve the understanding of the role of SCFAs in the pathogenesis of COPD. In addition, an important focus of future research should be to investigate the causal relationship between diet, gut microbiome structure, SCFAs production, and COPD progression.

## 4. Clinical Significance of Short-Chain Fatty Acids in the Pathogenesis of COPD

The presence of SCFAs in the sputum confirms the connection between the lungs and the intestine [82]. SCFAs are thought to contribute to the maintenance of lung immunometabolic tone [74]. As already noted, SCFAs can act on various parts of the innate immune defenses of the lungs. In addition to the described anti-inflammatory effects, the protective role of butyrate and propionate may include the restoration and maintenance of the barrier function of damaged airway epithelium by increasing the expression of ZO-1 dense contact proteins [127]. This seems important given the development of airway epithelial barrier dysfunction and impaired tight cell contacts in smoking and COPD [128]. In this regard, the regulation of barrier function under the influence of SCFAs may have some clinical significance [127].

The results showed differences in the composition of the gut microbiome in COPD and in healthy individuals. This is consistent with lower overall levels of SCFAs in the stage III-IV COPD group than in patients with stage I-II COPD and in healthy individuals [129]. Experimental data indicate that transplantation of fecal microbiota to mice led to inflammation in the lungs and increased IL-1β and TNF-α in plasma. At the same time, additional exposure to smoke from the biomass led to an accelerated decrease in lung function, emphysematous changes, airway remodeling, and mucus hypersecretion. These changes were accompanied by higher levels of claudin 1, α smooth-muscle actin (α-SMA), neutrophil elastase (NE) and matrix metalloproteinase 2 (MMP-2), and MUC5AC. In addition, gut microbiota obtained from stage III-IV COPD patients decreased body weight in recipient mice [129]. It should be noted that another recent study did not show an association of gut microbial diversity with the severity of COPD [130].

**Figure 3:**
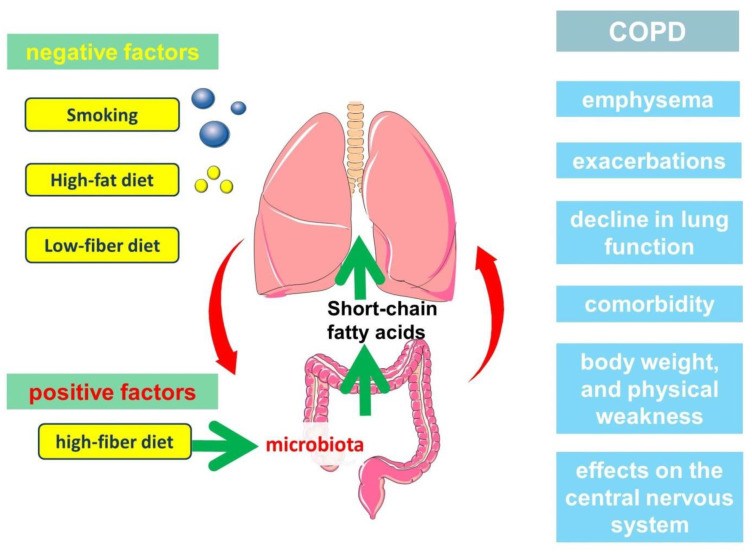
The participation of short-chain fatty acids in the pathogenesis of the clinically heterogeneous course of COPD.

Thus, SCFAs are an important part of the immunological axis linking the gut microbiota and the lungs, which has implications in the pathogenesis of COPD (Figure 3). In another study, patients with COPD with a stable moderately severe course of the disease had increased the total concentration of SCFAs in exhaled breath condensate [131]. Another study involving 38 COPD patients showed a decrease in acetate and an increase in the relative content of propionate, butyrate and branched-chain SCFAs in sputum and feces [132].

Patients with asthma showed a significant decrease in the total content of SCFAs in the feces, changes in the absolute concentrations of individual acids: acetate, propionate, butyrate, and changes in the total content of short-chain branched-chain fatty acids were also detected [133]. Different variations in the composition of SCFAs were shown, but changes in the metabolic profile were independent of the disease phenotype [133].

These and other data suggest that SCFA levels may be related to the pathogenesis of COPD, but the details of these relationships require clarification.

## 5. Clinical Significance of Short-Chain Fatty Acids in the Pathogenesis of COPD

COPD is a chronic respiratory disease characterized by progressive airflow limitation [134]. The rate of decline in lung function is individual and can vary widely from person to person. It should be noted that individual trajectory of COPD progression includes not only airflow limitation [135,136,137]. It is of great interest to phenotype patients according to the peculiarities of the clinical picture [138,139]. It should be noted that, to date, discussions about COPD phenotypes have not led to a generally accepted understanding of clinical variants, the separation of which would help to improve approaches to the management of patients [140,141].

The concept of COPD phenotypes has deep historical roots and goes back to the recognition of the two main components of the disease, such as emphysema and chronic bronchitis. They were described long before the term COPD itself appeared and were well known to clinicians.

### 5.1. Role of Short-Chain Fatty Acids in the Development of Emphysema

Emphysema is an important clinical phenotype of COPD. Numerous studies are devoted to the analysis of pathogenetic mechanisms of its development, among which of interest are the works studying the relationship of emphysema with nutrition. It is important to know about the development of emphysema during prolonged starvation, which was shown in the study of Warsaw ghetto prisoners, as well as observations in patients with anorexia nervosa [142,143]. These data, reinforced by experimental animal models, confirmed the link between nutrient deficiency and alveolar tissue destruction that leads to emphysema [144]. There are various explanations that justify these connections. Interestingly, anorexia nervosa is characterized by a decrease in intestinal microbial diversity, which is associated with the production of SCFAs, including butyrate and propionate [145,146].

A high-fiber diet has been shown to help prevent the progression of emphysema caused by cigarette smoke exposure. This is because a high-fiber diet changes the composition of the gut microbial community, resulting in increased production of SCFAs (acetate, propionate, and butyrate). SCFAs attenuated the pathological changes associated with the progression of emphysema and influenced the inflammatory response caused by cigarette smoke exposure [147]. Mice with emphysema that received fermentable fiber (pectin) were shown to have less inflammation than mice with emphysema that received nonfermentable fiber such as cellulose. This corresponded to lower concentrations of acetate, propionate, and butyrate in the emphysema group compared with the other groups. At the same time, macrophage and neutrophil counts were lower in the high fiber (cellulose and pectin) group than in the emphysema group [147]. In addition, inflammatory cytokine levels were lower in the high-fiber (cellulose and pectin) diet. In another study, butyrate was shown to reduce hypoxia-induced accumulation of alveolar (mainly CD68+) and interstitial (CD68+ and CD163+) macrophages in rat lungs [148].

The key events leading to emphysema are considered to be interalveolar septal avascularization, which is associated with the apoptosis of endothelial cells and alveolar epithelial cells [149]. In this regard, the information about butyrate participation in endothelial cell function is of interest. The anti-inflammatory properties of butyrate are in part related to its effect on the activation of NF-kB and PPARalpha and the associated expression of VCAM-1 and ICAM-1 [85,148]. In addition, butyrate inhibited the activation of endothelial NRLP3 inflammasome in endothelial cells [150]. Butyrate has also been shown to be involved in the inhibition of angiogenesis through HIF-1α as well as through the inhibition of vascular endothelial growth factor (VEGF) and cyclooxygenase-2 (COX-2) [148,151,152]. Butyrate has also been shown to enhance the expression of tight junction proteins in pulmonary microvascular endothelial cells, affecting endothelial barrier function [148].

The effects of butyrate in endothelial cells are also related to NO production [153]. It has been shown that butyrate and acetate, can improve AngII-induced endothelial dysfunction through increased NO bioavailability [154]. The reduction of reactive oxygen species (ROS) levels in the vascular wall and the subsequent prevention of NO inactivation represent a key mechanism through which SCFAs act on endothelial function [154]. These findings are of interest given the frequent association of COPD with atherosclerosis.

Thus, the production of SCFAs by the intestinal microbiota may be one possible mechanism in the prevention of emphysema. It was found that a serum peptide-based enteral diet can suppress elastase-induced emphysema in mice by altering SCFAs levels in the cecum [155]. Modulation of the gut microbiota by prebiotics and transplantation of fecal microbiota from a high-fiber diet altered the composition of the gut microbiota, attenuating smoking-induced emphysema [156]. This was associated with a decrease in local and systemic inflammation through production of SCFAs, which protect against alveolar destruction and cellular apoptosis. The levels of IL-6 and IFN-γ in bronchoalveolar lavage fluid (BALF) were lowest in the high-fiber diet group, and the local concentration of SCFAs was markedly higher in mice with emphysema following fecal microbiota transplantation and the high-fiber diet than in mice with emphysema [156]. And the high-fiber diet had a more protective effect against emphysema than the high-protein diet [156].

Experimental data showed that Firmicutes species were most dominant in samples from the feces of emphysematous mice. Transplantation of fecal microbiota from a high-fiber diet resulted in increased abundance of Bacteroides phylum, decreasing the Firmicutes/Bacteroides ratio [156].

Thus, the development of emphysema, a key phenotype of COPD, may to some extent be related to the role of SCFAs in the maintenance of lung immune function. It should be noted that the pathophysiology of emphysema includes many known mechanisms unrelated to SCFAs, many of whose links are currently the subject of research. That said, the possible role of SCFAs in the pathophysiology of emphysema in COPD is a promising area for future research that could provide answers to some questions related to the clinical efficacy of nutritional support for patients.

### 5.2. Role of Short-Chain Fatty Acids in COPD Exacerbations

An important characteristic of the natural course of COPD is the frequency and severity of exacerbations. COPD exacerbations make a significant contribution to the clinical picture of COPD. The results of numerous studies suggest that the frequency of exacerbations is associated with a more rapid decrease in FEV1 and an unfavorable prognosis [157,158,159]. Given these facts, some authors propose to consider high exacerbation frequency as a separate phenotype [158,160,161]. Infectious exacerbations of COPD are associated with disturbances in the structure of microbiota in the bronchi [162]. Colonization of the bronchi by microorganisms is necessary to maintain the immunological tone of the lungs. The available data suggest certain links between the intestinal and lung microbiome [163]. The respiratory tract microbiome can be supplemented with microorganisms from the gastrointestinal tract, which is important.

It is important to note that diet can affect not only the gut microflora, but also the respiratory tract microbiota [163,164]. SCFAs can have a direct effect on microorganisms as well as affecting their virulence [165]. Interestingly, high concentrations of SCFAs caused significant inhibition of Pseudomonas aeruginosa growth, which was enhanced at lower pH. At the same time, low concentrations of SCFAs resulted in enhanced bacterial growth [82]. Meanwhile, the administration of prebiotics in the form of oligosaccharides can modulate the immune and inflammatory response and outcome of pulmonary Pseudomonas aeruginosa infection in C57BL/6 mice through effects on the gut microbiota [166].

In addition, the structure of the gut and lung microbiota was shown to be dynamic, with changes associated with exacerbations. [167]. COPD exacerbations were characterized by a decrease in the relative content of Firmicutes and Actinobacteria, and an increase in Bacteroidetes and Proteobacteria compared with stable COPD and nonsmokers [168].

It should be noted that antibiotic therapy used to treat COPD exacerbations can have a serious impact on the structure of the gut microbiome and have long-term implications for metabolic and immunologic health [169]. In addition, macrolide use in childhood has also been shown to be associated with long-term changes in gut microbiota composition and function. These changes are associated with metabolic disease and obesity, and may also affect the development of the immune system, leading to respiratory hypersensitivity [169,170].

### 5.3. Role of Short-Chain Fatty Acids in the Decline of Lung Function

The rate at which lung function decreases is important in assessing the prognosis of patients with COPD. Given that bronchial obstruction is irreversible, progressive decline in lung function is associated with multiple systemic effects and the increased severity of comorbid conditions. Rapid progressive decline in lung function in COPD is suggested by some authors to be a separate phenotype, given its relationship with the prognosis of the disease. Moreover, the rate of decline in lung function is associated with many factors, including the frequency of exacerbations. Interestingly, nutrition can also influence lung function. The high intake of sweets, oils, fat, and coffee has been shown to be negatively associated with lung function, including FEV1/FVC, and has been associated with an increased prevalence of COPD in men [20,171,172,173,174]. In contrast, high intake of fruits and vegetables, fatty fish, and low-fat foods was negatively associated with a diagnosis of COPD [175,176,177].

Interestingly, however, the rate of decline in lung function may be related to impaired biodiversity of the gut microflora. It has been shown that a rate of decrease in FEV1 of more than 40 mL/year corresponded to a greater decrease in bacterial diversity in the gut [178]. In a one-year follow-up, it was shown that at the phylum level, Firmicutes were more abundant in the group with decreased lung function, whereas Bacteroidetes were more abundant when lung function was virtually unreduced [178]. This corresponded to a more unstable gut microbiota in the reduced group than in the control group. The relative abundance of Prevotella was also shown to decrease after 1 year in the reduction group, which may indicate a subtype of COPD in which Prevotella abundance may be related to the rate of decline in lung function [178]. It has been previously noted that Prevotella may be associated with inflammatory disease [96].

Another study showed a link between differences in the bacterial community structure of the gut and sensitization to aeroallergens and lung function (FEV1) in asthma [179]. These data are of interest given the presence of a Asthma-COPD overlap syndrome (ACOS), which is important because it has some diagnostic difficulties in real clinical practice. Consumption of a single dose of soluble fiber (3.5 g of inulin) in patients with asthma was shown to result in a significant reduction in airway inflammation and improvement in lung function (FEV1 and FEV1/FVC). This effect was characterized by a decrease in the total number of sputum cells, including neutrophils, macrophages, and lymphocytes, and a decrease in IL-8 in sputum and exhaled NO. This was consistent with the significantly increased expression of GPR41 and GPR43 genes in sputum cells [180].

Increased circulating levels of SCFAs in mice fed a high-fiber diet protected against allergic inflammation in the lungs. In contrast, a low-fiber diet increased allergic airway disease by reducing SCFAs levels. In addition, elevated levels of circulating SCFAs were found to protect against allergic inflammation in the lungs. Propionate, by affecting the maturation of lung dendritic cells, promoted their high phagocytic capacity but impaired their ability to stimulate the effector function of T helper type 2 (TH2) cells [164].

### 5.4. Short-Chain Fatty Acids, Body Weight, and Physical Frailty Phenotype

The role of diet as an important factor modifying the course of COPD has been the subject of numerous studies. Many of these studies have focused on the role of polyunsaturated fatty acids (PUFAs), especially ω-3 fatty acids [181,182]. Their influence on disease progression and prognosis has been analyzed, which is related to the involvement of both PUFAs themselves and their metabolites in the regulation of inflammation and resolution of inflammation.

Analysis of the role of dietary fiber is another important area that has been shown to be clearly related to the course of COPD. A population-based prospective cohort of 35 339 Swedish women evaluated the association between baseline and long-term dietary fiber intake and COPD risk. The results of this study showed that high fiber intake is an important modifiable factor that may reduce the risk of COPD primarily in current and former smokers [183].

The relationship between SCFAs production and body weight is of interest, given its relevance to the pattern of COPD course. Fecal SCFAs concentrations were shown to be higher in overweight people than in lean people (80±6 vs 56±6 mmol/kg, *p* = 0.02). It was also found that overweight individuals may absorb more SCFA from the large intestine [184]. Another study showed that overweight or obese individuals had higher levels of fecal acetate, propionate, butyrate, and valerate compared to lean subjects [185].

GPR43 is known to play an important role in white adipose tissue (WAT) [186]. In an experiment in mice, SCFAs have been shown to suppress insulin-mediated fat accumulation through GPR43 activation. In mice attempting a high-fat diet, SCFAs levels in feces and plasma acetate were decreased, while GPR43 expression in the WAT was markedly higher compared to mice receiving a normal diet [186]. This may be part of the mechanism of energy balance regulation, which includes suppression of excess energy accumulation and increased fat consumption. In this mechanism, GPR43 may function as an energy sensor that promotes the use of excess energy in other tissues rather than to store it as fat in adipose tissue [186].

The physical frailty phenotype is less well known, but its clinical and prognostic significance is beyond doubt [141,187,188]. Physical frailty is a complex syndrome characterized by loss of physiological and cognitive reserve [189,190]. Impaired muscle metabolism and depletion of muscle mass is an important clinical characteristic of COPD, especially in older individuals. Reduced physical activity due to physical frailty is suggested to be considered as a COPD phenotype associated with unfavorable prognosis. Of considerable interest is the information that the species diversity of the fecal microbiota of the elderly is inversely related to physical performance and the Rockwood clinical frailty scale [191,192]. The composition of the gut microbiota has been shown to be closely related to the development of physical frailty in older adults [191]. A key link that may link gut microbiota structure to skeletal muscle function is SCFAs [193,194]. SCFAs can act as ligands for FFAR2 and FFAR3 in skeletal muscle cells, regulating metabolic pathways related to glucose uptake and metabolism as well as modulating mitochondrial biogenesis [195].

Through the inhibition of histone deacetylase, butyrate can lead to the prevention of apoptosis in muscle. Butyrate can affect muscle metabolism, including improving glucose metabolism and increasing enzymes involved in oxidative metabolism, thus preventing age-related muscle atrophy in mice [196]. Butyrate has been shown to increase the muscle fiber cross-sectional area and reduce intramuscular fat accumulation in older mice [196].

Thus, SCFAs may be involved in numerous extrapulmonary effects related to effects on metabolism in tissues, which is of great research and clinical interest.

### 5.5. Short-Chain Fatty Acids, the Central Nervous System, and the COPD Emotional Fragility Phenotype

Emotional lability is increasingly recognized as a phenotype of COPD because of its significant impact on treatment efficacy. Anxiety and depression are known to be associated with decreased quality of life, as well as increased hospitalizations and mortality. The data accumulated to date have strengthened our understanding of the links between COPD and these disorders.

Of great interest is the evidence of the influence of gut microbiota on the central nervous system through SCFA_S_, due to their several neuroactive properties. The exact mechanisms of these connections are still a subject for research. SCFAs have been shown to affect several neurological and mental diseases and behavioral processes. Their involvement in neuronal development, microglia maturation and the release of synaptic neurotransmitters is also known [43,197,198,199,200]. The involvement of SCFAs in neuroimmune processes in neurodegenerative diseases is important [200].

Short-chain fatty acids are involved in the onset of depression, which has been shown in macaques [201]. And it was found that in addition to plasma concentrations, some SCFAs (acetic acid, propanedioic acid, and butyric acid) are also impaired in macaque cerebrospinal fluid in a natural depression model. Butyrate levels differ significantly in both serum and liquor samples from these macaques [201]. These results are consistent with evidence of lower fecal SCFAs concentrations in depressed patients than in controls [202].

Another study showed that acetate production by the rodent intestinal microbiota on a high-fat diet leads to effects on the central nervous system. This is due to activation of the parasympathetic nervous system, resulting in the increased secretion of ghrelin, which promotes hyperphagia, and increased energy deposition in the form of fat due to increased glucose-stimulated insulin secretion [203]. At the same time, FFAR3, which is activated mainly by propionate and butyrate, regulates sympathetic activity by sensing nutritional status, thereby maintaining the body’s energy homeostasis [38]. In addition, butyrate has an antidepressant-like effect in mouse models and also improves cognitive abilities in rats [204,205,206]. These data are of clinical interest and are an important topic for further research.

Thus, in accordance with modern concepts, COPD is considered to be not only as a lung disease, but also from the position of its numerous systemic effects. Moreover, SCFAs may be involved in the regulation of many links related to the development and progression of COPD. In this regard, the complex influence of SCFAs on various pulmonary and extrapulmonary effects of COPD can be assumed.

## 6. Conclusion

COPD is a clinically heterogeneous disease characterized by the development and progressive restriction of airflow due to chronic inflammation in the bronchi. Despite the known etiological factor, cigarette smoking, many aspects of COPD pathogenesis are still unknown. The causes of heterogeneity of the COPD course, which is associated with individual trajectories of progression and prognosis, remain a subject of discussion. Nutrition has a marked effect on the course of the disease. Indeed, patients who are severely underweight are at greatest risk for adverse COPD outcomes. The development of emphysema is also related to dietary patterns. Non-digestible carbohydrates, such as fiber, have been shown to favorably influence prognosis. Although these fibers are inaccessible to human digestive enzymes, they are actively metabolized by intestinal microflora. The products of enzymatic activity are SCFAs, which are an important link in the gut-lung immune axis. Short-chain fatty acids demonstrate a variety of functions in the regulation of inflammation and may play an important role in the clinical picture of COPD. SCFA production depends on the nature of the diet and the structure of the microflora. In this regard, dietary modification to include non-digestible fibers in the diet is seen as an important therapeutic tool that can affect not only the course of the disease, but also its outcome.

It should be noted that many of the effects of a diet containing dietary fiber may be related not only to the microbial fermentation of this fiber in the gut and SCFAs production, but also to various other dietary components, including micronutrients and vitamins. These findings are reflected in numerous studies that emphasize the importance of individual nutritional components [207,208,209]. Given the contribution of other nutritional components, it would not be very correct to link the clinical features of the course of COPD solely with SCFAs. It can be stated that the problems of diet in the natural history of COPD are far from being solved and require new research. In addition, individual trajectories of the natural history of COPD are shaped by many external and internal factors, a simplified understanding of which will not contribute to the interpretation of research results and improve approaches to the management of patients.

Indeed, many questions concerning both the pathophysiology of COPD and the involvement of SCFAs in these processes remain unanswered to date. Importantly, COPD patients are not a clinically homogeneous group, which requires a differentiated approach in the evaluation of research findings. The molecular mechanisms exhibited by SCFAs require new experimental and clinical confirmations. In this regard, investigation of the role of SCFAs in the clinically heterogeneous course of COPD may be a promising area for future research. These data will help to broaden the understanding of the pathophysiological mechanisms and their impairments that are associated with COPD phenotypes. A better understanding of these mechanisms will enhance the development of more effective therapeutic intervention programs that will be better adapted to individual disease course trajectories.

## Data Availability

Not applicable.
